# Effective Treatment of Nonalcoholic Fatty Liver Disease Using a Community-Based Weight Management Program

**DOI:** 10.7759/cureus.16709

**Published:** 2021-07-29

**Authors:** Fadi Hawa, Mark Gladshteyn, Sejal V Gunaratnam, Caleb Scheidel, Paul M Corsello, Stephen H Berger, Naresh T Gunaratnam

**Affiliations:** 1 Internal Medicine, St. Joseph Mercy Ann Arbor Hospital, Ann Arbor, USA; 2 Internal Medicine/Gastroenterology and Hepatology, University of Michigan, Ann Arbor, USA; 3 Internal Medicine/Gastroenterology and Hepatology, Huron Gastroenterology Associates, St. Joseph Mercy Ann Arbor Hospital, Ann Arbor, USA; 4 Biostatistics, St. Joseph Mercy Ann Arbor Hospital, Ann Arbor, USA

**Keywords:** hepatic steatosis, nonalcoholic fatty liver disease (nafld), obesity treatment, fatty liver treatment, nafld and obesity, obesity-related illnesses, plant-based diet, mediterranean diet, community based, lifestyle intervention

## Abstract

Introduction

Obesity-related gastrointestinal disorders including nonalcoholic fatty liver disease (NAFLD) and gastroesophageal reflux disease (GERD) are more frequent and usually present earlier than type 2 diabetes mellitus (T2DM) and cardiovascular disorders. This provides an opportunity for the gastroenterologist to intervene early with an effective weight-loss therapy. We evaluated the outcomes of a multifaceted, community-based gastroenterologist-supervised weight management program compared with patient-directed weight-loss efforts after physician advice. The program is aimed at achieving a 10% total body weight (TBW) loss at three months, a known determinant for NAFLD regression.

Methods

This is a retrospective pre- and post-intervention study of NAFLD patients, who participated in a medically supervised weight management program in the period between May 2017 and May 2019. The program is comprised of a very-low-calorie (800 kcal/day) meal replacement diet, a recommended medical fitness program, and weekly behavioral support groups. Patients are followed on monthly basis and slowly transitioned to a whole food plant-based or Mediterranean diet after three months of participation. Patients’ weight trends driven by self-directed efforts to lose weight after physician advice were collected based on historical data up to two years prior to program participation. The primary outcome was defined as percentage TBW loss at three months under medical supervision (post-intervention) compared with patient-directed weight-loss efforts (pre-intervention). The secondary outcomes included percentage TBW loss in relation to behavioral support group attendance and improvement in GERD and T2DM disease status after program participation. Linear mixed and linear regression models were used to assess for a statistically significant difference in percentage TBW loss. Statistical significance was defined as p < 0.05.

Results

A total of 114 NAFLD patients (mean age 55 years, mean BMI 39 kg/m^2^, 77 females, and 37 males) completed at least three months of follow-up and were included in the study. Of those, 89 patients had a documented three-month office visit. At three months, 65% of patients had lost at least 10% of their TBW. Percentage TBW loss under medical supervision was noted to be significantly higher and occurred at a faster rate over three months when compared with patient-directed efforts after physician advice (p < 0.001). Patients who attended the behavioral support groups ≥ 50% of the time had a 3% higher TBW loss at three months compared with patients who attended <50% of the time (p = 0.006). Approximately, 52% of patients with GERD and 38% of patients with T2DM had symptoms improvement and/or medication reduction at their three-month follow-up visit.

Conclusion

A multifaceted, community-based, gastroenterologist supervised weight management program is effective in achieving a clinically significant TBW loss of at least 10% within three months of participation. This weight loss was greater and occurred at a faster rate when compared with patient-directed efforts. Additionally, improvement in GERD and T2DM disease status was noted in 52% and 38% of patients with these conditions, respectively. Further community-based studies of a larger scale are needed to determine the sustainability of this weight loss over one year.

## Introduction

Obesity is a chronic, relapsing, and multifactorial disease, and it is the second leading cause of preventable death in the United States (US), closely behind tobacco use [1]. The obesity epidemic continues to be on the rise, posing a high burden on the population health and economy, with estimated annual medical expenses approaching 150 billion dollars [2]. It is estimated that by 2030, more than half of the US population will be considered obese [3]. In addition to being associated with numerous pathological conditions including cardiovascular disease (CVD) and type 2 diabetes mellitus (T2DM), obesity also exerts a significant toll on gastrointestinal (GI) disorders [4]. Obesity-related GI disorders such as nonalcoholic fatty liver disease (NAFLD) and gastroesophageal reflux disease (GERD) are frequently encountered in gastroenterology clinics [5]. These disorders are considered more frequent and present earlier than CVD and T2DM, thus providing an opportunity for gastroenterologists to intervene early with an effective weight loss therapy [5].

NAFLD is the most prevalent cause of chronic liver disease, affecting 25% of the world population and over 30% of the US population [6]. The increasing prevalence of NAFLD and its bidirectional relationship with CVD and components of the metabolic syndrome pose a significant economic burden with over $103 billion per year in direct costs and about $908 billion 10-year economic burdens for the management of disease-related complications [6,7]. Lifestyle modifications including weight loss and physical activity remain the cornerstone for the management of NAFLD [4,6]. However, given the complexity of obesity management, patient-directed efforts to lose weight after physician advice are usually unsuccessful, evidenced by the increasing prevalence of obesity [8]. Therefore, a structured medically supervised weight management program with regular follow-up visits is likely to be more effective [8]. Multiple randomized controlled trials have demonstrated the effectiveness of structured weight management programs combined with regular physical activity in the management of obesity-related gastrointestinal disorders, namely, NAFLD and GERD [9,10]. These interventions may be achievable in a controlled trial setting. However, they are considered challenging in a community-based clinical setting due to the lack of formal training of healthcare providers in effective weight loss methods [10,11].

The American Gastroenterological Association (AGA) developed practice guidance entitled “POWER: Practice Guide on Obesity and Weight Management,” which introduces gastroenterologists to effective obesity management in their routine practices [5]. This practice guide was used in designing the weight management program at our community-based gastroenterology practice [12]. The program is comprised of a multiphasic dietary intervention, a recommended medical fitness program, and weekly behavioral support groups. The main goal of the program is to achieve a 10% total body weight (TBW) loss at three months, which is a known determinant for NAFLD regression [13]. We hypothesized that a multifaceted, community-based, gastroenterologist-supervised weight management program is a more effective weight loss strategy than patient-directed weight loss efforts after physician advice. This article was previously presented as a meeting abstract at the 2020 Digestive Disease Week Annual Scientific Meeting on May 2, 2020 [14].

## Materials and methods

Study design and population

**Figure 1:**
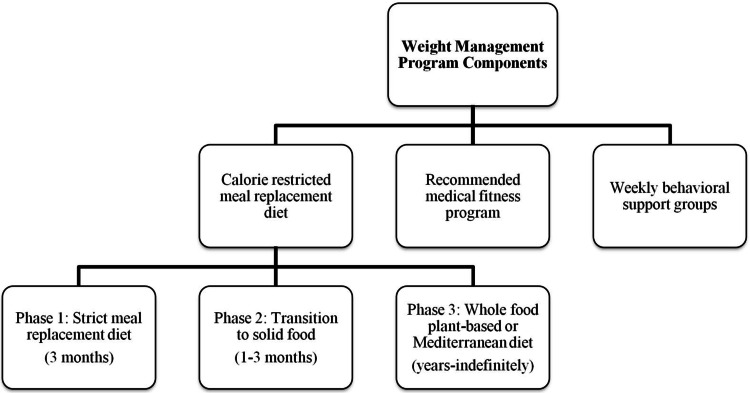
Weight management program components

We conducted a retrospective pre- and post-intervention study within the same cohort of patients with a primary diagnosis of NAFLD or NAFLD with GERD, who joined the weight management program for obesity-related GI disorders in the period between May 2017 and May 2019. The pre-intervention period reflects the patients' weight trends driven by self-directed efforts to lose weight prior to program participation, and the post-intervention period reflects those patients' weight trends under physician supervision after program participation. The weight management program is conducted at Huron Gastroenterology Associates, a community-based practice located in Ann Arbor, Michigan. The weight management team consists of three gastroenterologists, three physician assistants, a weight loss navigator, a medical assistant/data analyst, a registered dietician, and a registered nurse educator. The program is a three-phase dietary intervention; phase 1 consists of a strict very-low-calorie meal replacement diet for three months (Optifast, Nestle Health Sciences, Florham Park, NJ, USA; nutritional content (800 kcal/day): fat 19.4% kcal; carbohydrate 43.4% kcal; fiber 3.5% kcal; and protein 33.7% kcal), phase 2 is a slow transition to solid foods, and phase 3 is a complete transition to whole food - either plant-based or Mediterranean diet. The program also includes weekly behavioral support groups, focusing on patient empowerment, mindfulness, dietary education, and lifestyle modification strategies along with access to a recommended medical fitness program (Figure 1).

Details on the development and specifics of the program components have been previously published [12]. Eligible patients were obese adults (body mass index [BMI] ≥ 30 kg/m^2^) aged 18 years or older with a diagnosis of NAFLD, no history of excessive alcohol consumption (>21 drinks per week for men and >14 drinks per week for women over a two-year period), or evidence of other causes of chronic liver disease [15]. Hepatic steatosis index [HSI = 8 x (alanine aminotransferase/aspartate aminotransferase ratio) + BMI (+2, if female; +2, if diabetes mellitus)] of more than 36 and/or prior imaging studies showing hepatic steatosis (ultrasound, computed tomography scan, or transient elastography) were used to confirm NAFLD in our patient population [16]. GERD diagnosis was defined clinically by the presence of bothersome heartburn and/or regurgitation symptoms. Patients with NAFLD or NAFLD with GERD who participated in the program and completed at least three months of follow-up were included in the study. Patients who underwent intragastric balloon placement to promote weight loss were excluded from the study.

Study measurements

Manual data collection was obtained retrospectively through accessing the electronic medical records of patients participating in the weight management program. Data was then reviewed by a single investigator (M.G.) using the inclusion and exclusion criteria described above.

The following variables were collected for patients who met the inclusion criteria: general demographics (gender, age, and race/ethnicity), obesity-related GI disorders (NAFLD, NAFLD with GERD), medical comorbidities (T2DM, hypertension, and hyperlipidemia), weight-related variables (baseline weight [kg], BMI [kg/m^2^], weight at subsequent monthly office visits, and percentage TBW loss at three months), disease status at three months (defined by symptoms improvement and/or medication reduction for GERD, and medication reduction and/or improvement in glycosylated hemoglobin (HbA1c) for T2DM).

Behavioral support groups attendance was collected for each patient as a percentage (<50% attendance and ≥50% attendance), which is calculated by dividing the number of attended sessions over the total number of support groups offered to the patient since participation in the program. Compliance with the meal replacement diet was self-reported by patients during their regular follow-up visits.

Patients’ weight trends based on self-directed efforts to lose weight through diet modification and physical exercise after physician advice were collected by reviewing historical data for each patient included in the cohort up to two years prior to participation in the weight management program. A weight trend was established by at least two consecutive weight measurements approximately three or more months apart.

The primary outcome of the study was percentage TBW loss at the three months office visit (defined as a visit occurring after 9.5 weeks and less than 16 weeks after the baseline office visit in order to account for irregularly spaced monthly visits) compared with patient-directed efforts to lose weight after physician advice. The secondary outcomes included percentage TBW loss in relation to behavioral support group attendance and improvement in GERD and T2DM disease status after program participation.

Statistical analysis 

Descriptive statistics for baseline patient characteristics and three-month outcomes are reported using means and standard deviations for continuous variables, counts, and percentages for categorical variables. To assess if weight loss under medical supervision (post-intervention) was statistically significant when compared with patient-directed efforts after physician advice (pre-intervention), a linear mixed model was fit with a period (pre versus post) included as a fixed effect as well as time and intercept as random effects to account for the correlation of repeated measures within the same subject. A linear regression model with percent weight loss as the outcome and a covariate for support group attendance (≥50% versus <0% attendance) was fit to examine the effect of support group attendance on weight loss at three months. The model also controlled for age, baseline weight, BMI, and comorbidities. Statistical significance was defined as p < 0.05.

Ethical consideration

The study was approved by the St. Joseph Mercy Health System Institutional Review Board. Informed consent was waived given the retrospective nature of the study.

## Results

Between May 2017 and May 2019, 235 patients were offered participation in the weight management program. Of those, 208 patients presented for their baseline office visit, 36 patients (17.3%) opted not to pursue the program, and 58 patients (27.9%) did not complete three months of follow-up. A total of 114 patients with NAFLD or NAFLD with GERD who completed at least three months of follow-up were included in the study. Table 1 presents the patients’ baseline demographics and clinical characteristics.

**Table 1:** Baseline demographics and clinical characteristics of the study's patient population (n = 114) BMI: Body mass index; GERD: gastroesophageal reflux disease; NAFLD: non-alcoholic fatty liver disease; SD: standard deviation. ^€^Range denotes the lower and upper limit of the data distribution.

Baseline characteristics	Total sample (n = 114)
Demographics	
Age (years [SD])	55.6 (12.6)
Gender (female [%])	77 (67.5%)
Race/ethnicity (non-Hispanic White [%])	103 (90.3%)
Disease status	n (%)
NAFLD	114 (100%)
GERD	73 (64%)
Comorbidities	n (%)
Type 2 diabetes mellitus	33 (29%)
Hypertension	61 (53.5%)
Hyperlipidemia	60 (52.6%)
Obesity measures	kg (SD)
Weight (kg)	110.3 (20.7)
BMI (kg/m^2^)	38.7 (6.4)
Hepatic steatosis index	49.7 (7)
Follow-up period	Median (range^€^)
Weeks	26.9 (17.4-39.5)
Support group attendance (number of patients)	n (%)
< 50%	71 (62.3%)
≥ 50%	43 (37.7%)
Support group attendance (median percentage of attendance)	Median (range)
< 50%	2% (0%-22%)
≥ 50%	75% (62%-100%)

The mean baseline weight of the study participants was 110 ± 20.7 kg (BMI 38.7 ± 6.4 kg/m^2^), indicating a reasonable amount of variation in baseline weight among the participants. Approximately 90% of participants were non-Hispanic White, with the remaining participants declining to specify their race or ethnicity or being the lone participant in their respective race/ethnic group.

**Figure 2:**
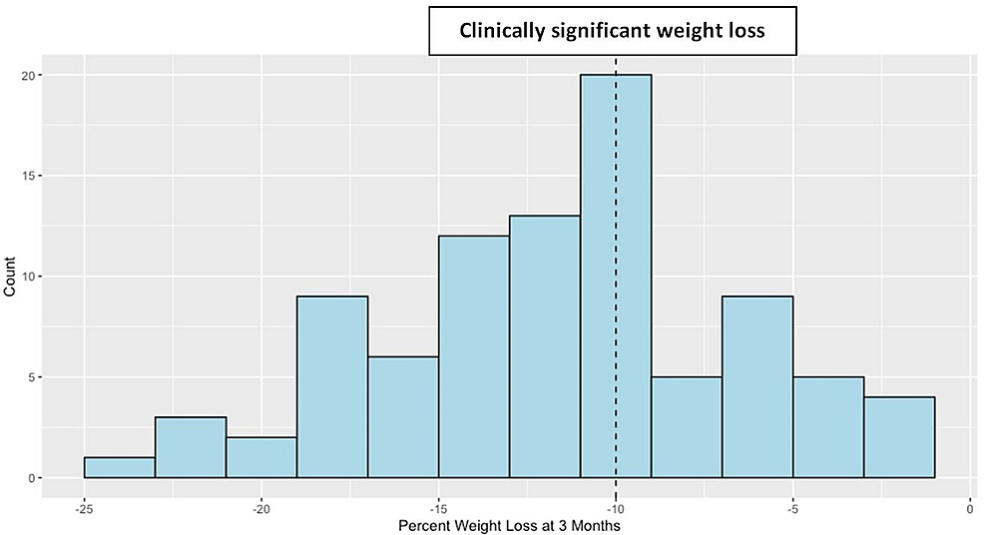
Histogram of percentage weight loss at three months (n = 89)

Monthly follow-up visits were noted to be irregularly spaced. Therefore, a valid three-month follow-up office visit was defined as a visit occurring after 9.5 weeks and less than 16 weeks after the baseline office visit. A total of 89 patients had a valid three-month office visit while the remaining 25 patients had data recorded either prior to 9.5 weeks or after 16 weeks of follow-up. Hence, those patients were not included in the primary analysis. Of the 89 patients, 65% lost at least 10% of their body weight with a mean percentage TBW loss of 11.6% (Figure 2).

Table 2 details the 89 patients’ baseline demographics, clinical characteristics, and percentage weight loss.

**Table 2:** Baseline demographics and clinical characteristics of the patients with a valid three months office visit (n = 89) BMI: Body mass index; GERD: gastroesophageal reflux disease; NAFLD: non-alcoholic fatty liver disease; SD: standard deviation. ^€^This column displays baseline data for the 89 patients who had a valid office visit at three months (between 9.5 weeks and 16 weeks). The remaining 25 patients had data recorded either prior to 9.5 weeks or after 16 weeks. ^ǁ^Non-responders: Patients with < 10% weight loss at three months. Responders: Patients with ≥10% weight loss at three months. ^§^p-values are comparing non-responders (<10% weight loss at three months) versus responders (≥10% weight loss at three months). ^£^Range denotes the lower and upper limit of the data distribution.

Baseline characteristics	Patients with a valid three months office visit^€^ (n = 89)	Non-responders (n = 31)^ǁ^	Responders (n = 58)^ǁ^	p-value^§^
Demographics				
Age (years [SD])	55.4 (13.2)	59 (11.7)	53.5 (13.7)	0.05
Gender (Female [%])	60 (67.4%)	22 (71%)	38 (65.5%)	0.78
Race/ethnicity (non-Hispanic White [%])	81 (91%)	29 (93.5%)	52 (89.7%)	0.70
Disease status	n (%)	n (%)	n (%)	
NAFLD	89 (100%)	31 (100%)	58 (100%)	-
GERD	56 (62.9%)	21 (67.7%)	35 (60.3%)	0.65
Comorbidities	n (%)	n (%)	n (%)	
Type 2 diabetes mellitus	23 (25.8%)	11 (35.4%)	12 (20.7%)	0.21
Hypertension	46 (51.7%)	23 (74.2%)	23 (39.7%)	0.004
Hyperlipidemia	46 (51.7%)	17 (54.8%)	29 (50%)	0.83
Obesity measures	kg (SD)	kg (SD)	kg (SD)	
Weight (kg)	109.8 (18.1)	106.2 (14.6)	111.7 (19.5)	0.14
BMI (kg/m^2^)	38.4 (5.5)	37.9 (5.6)	38.7 (5.5)	0.52
Hepatic steatosis index	49.3 (6.1)	48.6 (6.8)	49.7 (5.8)	0.45
Percentage weight loss at three months	-11.6 (5)	-6.5 (2.7)	-14.4 (3.6)	< 0.001
Follow-up period	Median (range^£^)	Median (range)	Median (range)	
Weeks	26.9 (15.6-39.5)	23.4 (13.9-38.3)	27.8 (17.9-40.2)	0.39
Support group attendance (number of patients)	n (%)	n (%)	n (%)	
< 50%	54 (60%)	22 (71%)	31 (53.5%)	0.17
≥ 50%	36 (40%)	9 (29%)	27 (46.6%)	0.17
Support group attendance (median percentage of attendance)	Median (range)	Median (range)	Median (range)	
< 50%	2% (0%-23.5%)	5% (2%-19%)	0% (0%-24.7%)	0.20
≥ 50%	75% (62%-100%)	75% (68%-90%)	79% (62%-100%)	0.39

Of patients who had GERD at baseline and had a three-month follow-up visit, 52% showed symptom improvement and/or medication reduction. In addition, medication reduction and/or improvement in HbA1c levels was noted in 38% of patients with T2DM. Patients who attended the behavioral support groups <50% of the time had a mean weight loss of 4.7 ± 2 kg at three months, compared with patients who attended ≥50% of the time who had a mean weight loss of 6 ± 2.2 kg. For each additional 0.45 kg in baseline weight, the estimated percentage TBW loss at three months increased by 0.05% (95% confidence interval [CI], -0.09 to -0.01; p = 0.02). Results from a linear regression model with percentage weight loss at three months as the outcome are presented in Table 3.

**Table 3:** Linear regression model with percentage weight loss at three months as the outcome BMI: Body mass index; CI: confidence interval; GERD: gastroesophageal reflux disease; SE: standard error. ^€^The model was fit to the subset of patients who had a valid three-month office visit (n = 89). ^§ ^<50% attendance is the reference group for the support group attendance.

Coefficient^€^	Estimate	SE	95% CI	p-value
Baseline BMI	0.20	0.15	(-0.09, 0.50)	0.17
Baseline age	-0.02	0.04	(-0.10, 0.06)	0.68
Baseline weight	-0.05	0.02	(-0.09, -0.01)	0.02
Baseline GERD	0.29	1.08	(-1.86, 2.43)	0.79
Baseline hypertension	2.09	1.13	(-0.16, 4.35)	0.07
Baseline type 2 diabetes mellitus	0.80	1.18	(-1.55, 3.14)	0.50
Baseline hyperlipidemia	0.65	1.01	(-1.36, 2.66)	0.52
Support group attendance ≥ 50%^§^	-2.98	1.07	(-5.11, -0.86)	0.006

The percentage TBW loss under medical supervision while participating in the weight management program (post-intervention) was significantly higher when compared with patient-directed efforts after physician advice (pre-intervention) (Beta = -1.3, 95% CI, -1.43 to -1.17; p < 0.001). Additionally, the rate of weight loss was faster in the post-intervention period compared with the pre-intervention period (Beta = -0.83, 95% CI, -0.99 to -0.67; p < 0.001) (Table 4).

**Table 4:** Linear mixed model with percentage weight loss as the outcome (pre- versus post-intervention) BMI, Body mass index; CI: confidence interval; SE: standard error; Var: variance. ^€^The model was fit to the subset of patients who had a valid three-month office visit (n = 89). ^§^Post-intervention is the period during which the patient is participating in the weight management program as opposed to pre-intervention (reference group), which reflects the period of patient-directed weight loss efforts prior to participation in the weight management program.

Term^€^	Estimate	SE	95% CI	p-value
Fixed effects				
Intercept	0.81	0.07	(0.68, 0.95)	< 0.001
Period (post-intervention)^§^	-1.3	0.06	(-1.43, -1.17)	< 0.001
Days since baseline visit	0.05	0.09	(-0.12, 0.22)	0.55
BMI	0.08	0.05	(-0.02, 0.18)	0.13
Age	0.02	0.03	(-0.03, 0.08)	0.41
Baseline weight	-0.11	0.05	(-0.28, -0.01)	0.03
Period (post-intervention) * days since baseline visit^§^	-0.83	0.08	(-0.99, -0.67)	< 0.001
Random effects				
Var (intercept)	0.14			
Var (time)	0.19			
Residual	0.29			

**Figure 3:**
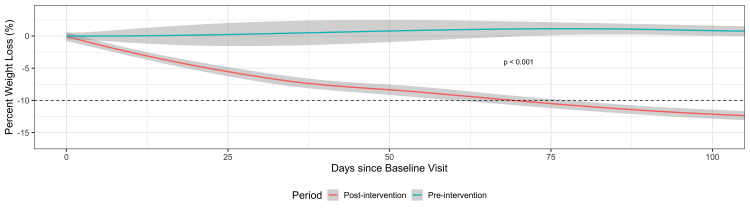
Percentage weight loss pre- and post-program participation (pre versus post-intervention)

Figure 3 shows the visual difference in the trend of percentage weight loss between the pre- and post-intervention periods.

**Figure 4:**
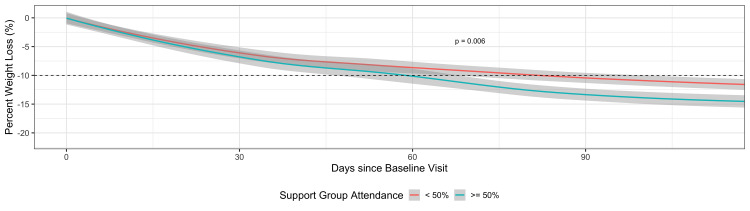
Patients' weight trends over time based on behavioral support group attendance

Those who attended the behavioral support groups < 50% of the time had a 3% lower TBW loss at three months compared with patients who attended support groups ≥ 50% of the time, holding all other covariates constant (95% CI, -5.11 to -0.86; p = 0.006). This difference is illustrated in Figure 4.

## Discussion

A multifaceted, community-based gastroenterologist-supervised weight management program with a behavioral intervention was effective in achieving a 10% TBW loss in 65% of participants at three months. Additionally, improvement in GERD and T2DM as demonstrated by symptom improvement and/or medication reduction was noted in 52% and 38% of patients with these conditions, respectively. Within the same cohort of patients, the program was significantly more effective at achieving weight loss than patient-directed efforts in the years preceding program participation. Participants who attended support groups ≥ 50% of the time had a 3% higher TBW loss at three months compared with patients who attended < 50% of the time. This study emphasizes the role of gastroenterologists in the multidisciplinary treatment of obesity in accordance with the AGA recommendations [5]. Patient-directed efforts during the previous years were ineffective in achieving any clinically significant weight loss, suggesting physician participation is a key determinant to success [8,11].

Gastroenterologists are not uniformly trained in effective weight loss techniques during their training [17]. Nonetheless, the providers participating in this study were able to facilitate effective weight loss in NAFLD patients, despite not having formal training in bariatric care. This was achieved using an algorithmic team-based approach, utilizing a dietician, a nurse educator, a data analyst, and a weight loss navigator. In addition, one of the program’s strengths lies in the ability to implement and reproduce this experience at other community-based gastroenterology practices [12].

The effectiveness of multifaceted behavioral weight management programs has been widely described in the literature [18-21]. However, much of the available evidence is derived from clinical trials that are conducted in a controlled setting under the supervision of experienced researchers at academic institutions. Our study suggests that incorporating this model in a community-based gastroenterology practice is achievable and is equally effective. Using a meal replacement product initially for up to three months was found to be effective in promoting patient compliance and eliminated the need for patients to abruptly change their eating patterns of prepared food. During the meal replacement period, intensive educational efforts were implemented to teach patients about the importance of fiber-rich, low glycemic index diets in preparation for transition to solid foods. Further emphasis was made on whole food - plant-based and Mediterranean diets - given the supportive evidence of their efficacy in long-term weight loss, improved glycemic control, and cardiovascular benefits [22,23].

Behavioral interventions have been consistently demonstrated as an important facet of any weight management program associated with long-term success as it improves dietary compliance and weight maintenance and prevents weight regain [24-27]. The behavioral and educational efforts in our program were delivered through face-to-face meetings, which were also live-streamed on Facebook® for patients who were unable to attend in person. The educational programs were also recorded and could be played back at the patient’s convenience. Findings from this study reinforce the importance of behavioral interventions as those patients who participated in the weekly behavioral support groups ≥ 50% of the time achieved a 3% higher TBW loss than those who did not.

Hepatic steatosis reversal can occur rapidly with lifestyle modifications. Reginato et al. evaluated the effects of short-term intensive lifestyle intervention on HSI in adults with obesity and/or T2DM [28]. This study showed that after three months of intensive lifestyle intervention, mean HSI significantly decreased from 45.7 to 43.4 (p < 0.001), while mean BMI decreased from 34 to 33.1 kg/m^2^ (p < 0.0001). Furthermore, Koutoukidis et al. performed a systematic review and meta-analysis to assess the association of weight loss interventions with changes in biomarkers of NAFLD [8]. The meta-analysis showed that more intensive weight loss interventions were significantly associated with a greater weight change (-3.61 kg; 95% CI, -5.11 to -2.12; I2 = 95%) when compared with no or lower intensity weight loss interventions. The aforementioned weight loss interventions were also associated with improvements in NAFLD biomarkers, including alanine aminotransferase (-9.81 U/L; 95% CI, -13.12 to -6.50), histologically or radiologically measured liver steatosis (standardized mean difference: -1.48; 95% CI, -2.27 to -0.70), histologic NAFLD activity score (-0.92; 95% CI, -1.75 to -0.09), and presence of nonalcoholic steatohepatitis (odds ratio, 0.14; 95% CI, 0.04-0.49). Most of the included studies were of short- to medium-term duration (median of six months). Based on these findings, the authors of the meta-analysis identified the need to change the clinical guidelines to recommend formal weight management programs for patients with NAFLD. The program instituted in our study may be an effective way to enact this recommendation.

Gastroenterologists have an opportunity to address obesity and provide effective therapy as it is considered a major modifiable cause of digestive tract diseases that routinely go unaddressed such as NAFLD and GERD [5]. Our study is consistent with the literature and demonstrates the effectiveness of a gastroenterologist-supervised weight management program that can be implemented at a community-based level and conducted by healthcare personnel in everyday routine practice. A recent study conducted by Malespin et al. found that only 32% of overweight or obese adults with NAFLD receiving usual care in the US achieved ≥5% TBW loss during a median follow-up of 39 months [29]. Therefore, the ability of our program to achieve a 10% TBW loss in 65% of patients who presented for their three-month follow-up office visit is encouraging, not to mention the additional benefits of the program demonstrated by the improvement in GERD and T2DM disease status in 52% and 38% of patients with these conditions, respectively. However, it should be noted that comorbid conditions such as hypertension and T2DM were relatively higher in patients who lost <10% TBW compared with those who lost ≥10% TBW. This observation suggests that pre-existing comorbidities may play a role in the weight management program's efficacy and patients' tendency to lose weight.

Our study’s main limitation is the irregularly spaced and non-standardized follow-up visits, which resulted in inconsistent data gathering and the inability to adequately assess our retention rates. However, this is an expected limitation of a weight management program implemented at a community-based practice, given the complex logistics of enforcing mandated follow-up visits in the absence of a clinical trial. We also noticed that some of our patients prefer less frequent follow-up visits once they have achieved their target weight loss goal. However, the majority of these patients are still in contact with our program either through attendance of behavioral support groups or through direct communication with our weight loss navigator via phone, text, or email, suggesting their desire for continued engagement with the program. Moreover, out of the 208 patients who presented for their baseline office visit, 36 patients (17.3%) opted not to pursue the program, and 58 patients (27.9%) did not complete three months of follow-up. This relatively high drop-out rate is another expected limitation of our community-based weight management program, which may be related to the very low-calorie diet (800 kcal/day) adopted in the initial phase of the program.

Other limitations of the study include the small sample size and the lack of a comparison group where only behavioral support is provided without meal replacement. In addition, the retrospective nature of the study limited our ability to collect dietary and exercise compliance data and biomarkers for NAFLD due to missing lab values. Furthermore, there are limits to the study's generalizability as the majority of patients were non-Hispanic White (90.3%), which is reflective of the practice's local community demographics. Finally, we were unable to assess our six months data by the end of the study period due to the inconsistent range of follow-up visit dates and missing data after the first three months. Hence, it is unclear if the results achieved at three months can be maintained for six to 12 months. However, anecdotal evidence suggests that patients who continue to engage in our educational programs for over a year are likely to maintain their weight loss. Quality improvement of the weight management program is ongoing and aims to address these limitations through utilizing telehealth services to ensure more regular follow-up visits that can assess long-term outcomes [30].

## Conclusions

A multifaceted, community-based, gastroenterologist-supervised weight management program is effective in addressing obesity-related gastrointestinal disorders including NAFLD and GERD, through achieving a clinically significant TBW loss of at least 10%. Incorporating a behavioral component into the program plays a vital adjunctive role in program success. Most importantly, the program can be implemented and reproduced at other community-based gastroenterology practices. Further larger scale and well-executed community-based studies are needed to determine the sustainability of this weight loss over one year.

## Appendices

Abbreviations

AGA: American gastroenterology association

CI: Confidence interval

CVD: Cardiovascular disease

GERD: Gastroesophageal reflux disease

GI: Gastrointestinal

HbA1c: Glycosylated hemoglobin

HSI: Hepatic steatosis index 

NAFLD: Non-alcoholic fatty liver disease

TBW: Total body weight

T2DM: Type 2 diabetes mellitus

US: United States
